# Probiotic therapy outcomes in body composition of children and adolescent with obesity, a nonrandomized controlled trial

**DOI:** 10.20945/2359-3997000000526

**Published:** 2022-10-11

**Authors:** Thaís Léo Pacheco Marcelo, Caroline Rosa Pellicciari, Thiago Olivetti Artioli, Dânae Braga Diamante Leiderman, Ana Lúcia Torloni Gradinar, Marcelo Mimica, Cristiane Kochi

**Affiliations:** 1 Irmandade Santa Casa de Misericórdia de São Paulo Departamento de Nutrição e Dietética São Paulo SP Brasil Departamento de Nutrição e Dietética, Irmandade Santa Casa de Misericórdia de São Paulo, São Paulo, SP, Brasil; 2 Irmandade da Santa Casa de Misericórdia de São Paulo Unidade de Endocrinologia Pediátrica Departamento de Pediatria São Paulo SP Brasil Departamento de Pediatria, Unidade de Endocrinologia Pediátrica, Irmandade da Santa Casa de Misericórdia de São Paulo, São Paulo, SP, Brasil; 3 Faculdade de Ciências Médicas da Santa Casa de São Paulo São Paulo SP Brasil Faculdade de Ciências Médicas da Santa Casa de São Paulo, São Paulo, SP, Brasil; 4 Faculdade de Ciências Médicas da Santa Casa de São Paulo Departamento de Microbiologia São Paulo SP Brasil Departamento de Microbiologia, Faculdade de Ciências Médicas da Santa Casa de São Paulo, São Paulo, SP, Brasil

**Keywords:** Pediatric obesity, probiotics, body composition, adolescent, microbiota

## Abstract

**Objective:**

The objective of this study was to evaluate the impact of probiotic supplementation therapy on anthropometric values and body composition of children and adolescent with obesity.

**Subjects and methods:**

This is a nonrandomized controlled, prospective, double-blind interventional clinical trial with primary data analysis. The sample comprised 44 pubertal children and adolescent (8-17 years old) with obesity. The patients were allocated to probiotic (Lactobacillus rhamnosus) or placebo group, with matching of gender and chronological age. Both groups received nutritional guidance, and were followed for six months. In all patients the anthropometric assessment was carried out by a nutritionist and data on weight, height and waist circumference (WC) were collected. Body composition was assessed using dual emission X-ray absorptiometry (DXA).

**Results:**

After six months, both groups had increased weight, height but reduced body index mass (BMI) standard deviation score, with no differences between groups. After the intervention, both groups showed a reduction in the percentage of total body fat and an increase in lean mass, but only the placebo group showed a reduction in the percentage of trunk fat. However, the variation in these parameters did not differ between groups.

**Conclusions:**

The probiotic group does not seem to have benefited from supplementation. However, we suggest that this reduction in BMI SDS in both groups may have occurred due to improvements in diet because of the nutritional advice given throughout the therapy. We concluded that supplementation with this strain of probiotic was not effective in promoting weight loss or improving the body composition of this population.

## INTRODUCTION

Obesity is a global public health challenge, both in developed and developing countries. In recent years, it has been shown that the development of obesity is also associated with intestinal microbiota changes and its modulation could play an important role in the treatment of obesity (1,2). One way to achieve this modulation of the intestinal microbiota may be through the use of probiotics, which could help the metabolism of the host, as certain strains have demonstrated beneficial effects on metabolism, favoring glucose homeostasis, interfering within inflammatory processes, reducing weight and body fat, and protecting against oxidative stress (3).

The dysbiotic microbiota of obesity has been widely discussed with both animal and human studies supporting the role of the use of probiotics in obesity (4). *Lactobacillus rhamnosus* GG (LGG) has been studied extensively in humans and experimental animals, due to its characteristic of being resistant to bile acids and its adherence to the intestinal epithelial layer. Thus, it is one of the most widely studied strains used in commercially available probiotics (5).

Wang and cols. (6) identified that *L. rhamnosus* LS-8 and *L. crustorum* were related to reduced weight and fat mass gain in rats that received a high fat and fructose diet, as well as to the attenuation of insulin resistance and inflammatory markers (6). In humans, descriptions of these effects are scarce and some of the studies show inconsistent results (7). Although studies evaluating probiotic therapy with *L. Rhamnosus* alone in children and adolescent with obesity are scarce, supplementation with probiotic mixture improved the liver profile in children with obesity and non-alcoholic fatty liver disease (8).

The objective of this study was, therefore, to evaluate the impact of probiotic supplementation therapy on anthropometric values and body composition of children and adolescent with obesity.

## SUBJECTS AND METHODS

### Trial design

This is a nonrandomized controlled, prospective, double-blind interventional clinical trial with primary data analysis carried out from April 2018 to October 2019. It was approved by the research ethics committee of our institution, under approval number 2.525.308 (CAAE: 82948218.3.0000.5479).

### Participants

The eligibility criteria were pubertal children and adolescent aged 8 to 17 years old of both genders who had been diagnosed with obesity according to their body mass index (BMI) z-scores as defined by the World Health Organization child growth standards (WHO, 2007) (9). The pubertal status was classified according to the Tanner criteria (10). All patients attended the pediatric endocrinology outpatient clinic of Santa Casa Hospital (philanthropic institution) in São Paulo, and they are from low income families. All parents or guardians and participants signed a consent form agreeing to their participation in the study. This was a convenience sample, and the exclusion criteria were patients with obesity secondary to genetic causes (syndromes), endocrine diseases (hypothyroidism, Cushing's syndrome, growth hormone deficiency), the presence of gastrointestinal diseases, infectious diseases at the time of examination, continuous use of glucocorticoids, medications that could interfere with weight, ingestion of yoghurts and products containing probiotics less than two weeks before the first exam, and patients who stopped using the probiotic during follow-up or did not perform the exams according to the study protocol.

Patients were allocated to each group matched by gender and chronological age. The allocation and distribution of the bottles were performed by an assistant physician of the staff and the main investigator was unaware of the vial contents. Therefore, nutritional guidance was given without knowing which group the patient belonged to.

### Interventions

**Figure 1:**
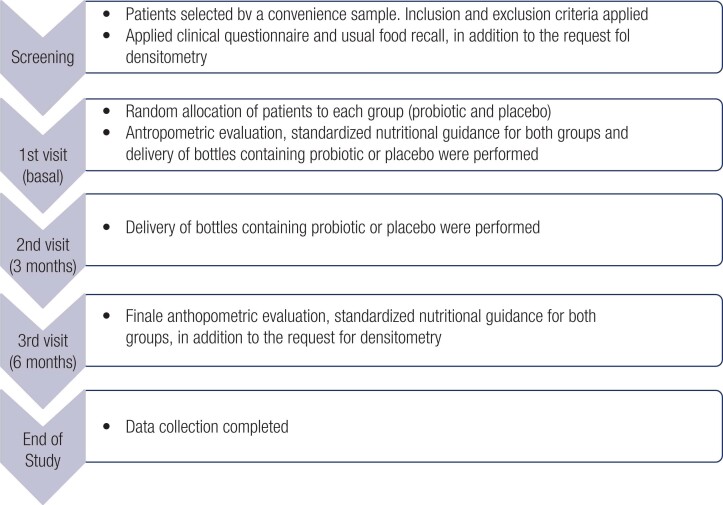
Flowchart of data collection process.

The flowchart of the data collection process is described in Figure 1. At the initial screening evaluation, an interview of usual dietary recall was applied to the patients, with the sole purpose of establishing the dietary profile of the studied population. The amounts of food consumed were recorded using a photographic manual of food quantification (11) which has photos of portions and forms of food, as well as photos of homemade measures, in order to standardize the quantification of food consumption.

The results of this assessment were then analyzed with the aid of Avanutri^®^ nutrition software. The reference values for macro and micronutrients were based on the Institute of Medicine's Dietary Reference Intakes (DRIs) (2001 and 2002) (12,13), considering the different ages of the studied group.

Patients were assessed over period of six months, with anthropometric assessments and standardized nutritional guidance at each visit (every three months). The nutritional guidelines provided in the consultations were the same for all patients in both groups and consisted of explaining the importance of fractioning meals, with food suggestions and information about nutritional composition, as well as recommendations on what to eat and what to avoid in the daily food routine, based on the *Dietary Guidelines for the Brazilian population* (14) and the *Dietary manual of the Brazilian Pediatric Society* (15).

For the probiotic group, a strain of *Lactobacillus rhamnosus* IAL 1883 (ATCC 7469) was used (supplied by Probac do Brasil and originally obtained from the Adolfo Lutz Institute). To prepare the strain, *Lactobacillus rhamnosus* was cultured on blood agar and chocolate agar, incubated at 35 ± 2 °C in an atmosphere of capnophilia for 24 hours. After growth on blood agar, the bacteria were inoculated in MRS broth, which was incubated at 35 ± 2 °C for 24 hours. After 24 hours, a broth sterility control was performed on blood agar and MacConkey agar, in order to guarantee the purity of the *Lactobacillus rhamnosus*. The probiotic was then prepared in a laminar flow hood with the inoculation of 1.9 microliters of *Lactobacillus rhamnosus* ATCC 7469 into flasks containing 90 mL of whey. The final concentration of the bacteria was 1.2 x 10^8^ CFU per mL of serum.

The placebo formulation consisted of 5% whey. This preparation was distributed in 95 mL flasks, capped and semi-autoclaved at 111 °C for 15 minutes. After cooling, they were closed completely.

Patients received two identical vials with 90 mL each (probiotic or placebo) per month and were instructed to keep the vials in a refrigerator (between 2 and 8 °C) and to ingest 5 mL of the solution once a day (upon waking). Regarding the viability of the probiotic, serial tests of the formulation were carried out, demonstrating continued growth of *Lactobacillus rhamnosus* in adequate concentrations for up to 90 days. In addition, conservation under refrigeration during the same period did not allow the growth of any other agent, including potentially pathogenic agents.

In both groups, the anthropometric assessment was carried out by a nutritionist and data on weight, height and waist circumference (WC) were collected. The weight was obtained using an electronic digital scale with a capacity of 150 kg and precision of 10 g (Filizola^®^ brand). Height was measured using a vertical stadiometer attached to the wall, with a length of 2 m, divided into centimeters and subdivided into millimeters. WC was obtained in duplicate, using an inelastic measuring tape according to conventional techniques (16). The average value was used as a reference.

BMI was calculated using the Anthro Plus software (WHO, 2007) (2). BMI and height values were expressed in Z-score (BMI SDS and height SDS) (16). The waist circumference by height ratio (WC/H) (17) was also calculated. Body composition was assessed using the dual emission X-ray absorptiometry (DXA, Lunar DPX-IQ, version 4.7e, Lunar Radiation Corporation, Madison, WI, USA) method at baseline and after six months of supplementation.

### Statistical methods

Statistical analysis was performed using the SigmaStat 3.5 for Windows program (Systat Software, Inc., San Jose, CA, USA). For analysis between different times of the same individual, the paired t-test or the Wilcoxon test was used. The comparison between the probiotic group and the placebo group was made using the Student t test for variables with normal distribution, and Mann-Whitney for variables that did not have a normal distribution, and p < 0.05 was considered statistically significant. Confidence intervals were constructed with 95% statistical confidence.

## RESULTS

**Figure 2:**
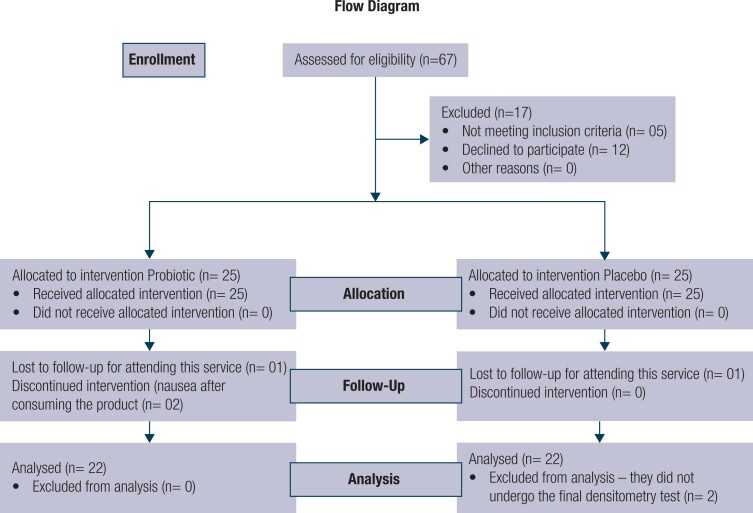
Results flow diagram.

Initially, of all the individuals with obesity attending the clinic, 67 patients agreed to take part and underwent screening. After applying the inclusion and exclusion criteria, 50 patients were selected, and 44 completed the study, adhering to the study protocol (Figure 2).

Of the total patients who completed the study, 56.8% (n = 25) were female and 43.2% (n = 19) male. The mean chronological age (SD) was similar in both groups, being 11.3 (2.0) years in the probiotic group and 10.6 (2.5) years in the placebo group.

The descriptive analysis of the dietary recall of the patients in both groups is shown in Table 1. There were no statistical differences between the groups in relation to consumption.

**Table 1:** Descriptive analysis of the consumption of macro and micronutrients in one day obtained at the initial dietary recall of the study in the probiotic and placebo groups, expressed as mean (SD)

Dietary Recall	Probiotic (n = 22)	Placebo (n = 22)
Calorie Intake	1974.2 (485.88)	1835.66 (461.86)
Carbohydrate Intake (%)	47.6 (8.57)	46.9 (6.73)
Protein Intake (%)	25.7 (5.38)	24.6 (5.00)
Total Fat Intake (%)	26.2 (5.87)	28.4 (5.75)
Saturated Fat (g)	15.2 (6.10)	16.1 (9.50)
Total Cholesterol Intake (mg)	312.1 (115.70)	265.8 (82.19)
Fiber intake (g)	16.6(6.92)	15.6 (6.02)
Calcium Intake (mg)	675.9 (576.08)	509.5 (335.99)
Sodium Intake (mg)	1805.5 (822.22)	1676.0 (667.44)

The analysis of macronutrient and micronutrient consumption showed that the intake of carbohydrates, proteins, total fats and sodium was within the recommended values for the age group in most patients. However, 91% (n = 20) of the probiotic group individuals and 86% (n = 19) in the placebo group had a total consumption of cholesterol above the recommended value, according to the recall. As for fiber consumption, we found that it was below the recommended in level in 100% (n = 22) of patients in the probiotic group, and 95.5% (n = 21) in the placebo group; Calcium consumption was also below the recommended level in 91% (n = 20) of the adolescent in the probiotic group, and 86% (n = 19) in the placebo group. The percentage of inadequate intake of each nutrient analyzed did not differ between groups (z test, p > 0.05).

The anthropometric characteristics at baseline and after the intervention of each group are described in Table 2. At the beginning of the study (baseline), there were no statistically significant differences between groups.

**Table 2:** Comparison between Probiotic and Placebo groups in relation to sex, age, height, BMI, BMI SDS abdominal circumference

	PROBIOTIC (n = 22)			PLACEBO (n = 22)		
Sex F:M	12:10			13:09		
Age	11.3 (2.0)			10.6 (2.5)		
	Basal	6 months	p-value	Basal	6 months	p-value
Weight (kg)	72.8 (20.5)	77.4 (20.78)	**P = <0,001** *	73.89 (18.1)	77.3 (19.0)	P = <0,001*
Delta Weight (kg)	5 (3.1-6.6)			4.55 (1.2-6.0)*		P = 0,255**
Height (cm)	152.9 (10.9)	157.2 (10.3)	**P = <0,001** *	155,2 (11,0)	158,4 (10,6)	**P = 0,001** *
Delta Height (cm)	4.35 (1.98)			3.15 (1.84)*		**P = 0.0438**
BMI (kg/m²)	28.9 (27.1- 35.9)	30.0 (26.6-35.6)	P = 0,570*	30,0 (5,1)	30,4 (5,3)	P = 0,256*
Delta BMI (kg/m²)	0.1 (-0.7-1.4)			−0.05 (-1.0-1.3)		P = 0,991**
BMI SDS	3.4 (2.8-3.7)	3.1 (2.8-3.5)	**P = 0,016** **	2,9 (0,6)	2,8 (0,6)	P = 0,060*
Delta BMI SDS	−0.24 (-0.32-0.04)			−0.05 (-0.23-0.08)		P = 0,270**
Waist Circumference (cm)	96.8 (11.4)	98.7 (12.8)	P = 0,113*	93,7 (13,0)	95,9 (13,9)	**P = 0,024** *
Delta Waist Circumference	1.89 (5.36)			2.15 (4.17)		P = 0,854*
Waist Circumference/Height (WC/H)	0.63 (0.04)	0.61 (0.05)	P = 0,141*	0,60 (0,07)	0,60 (0,07)	P = 0,717**
Delta WC/H	−0.01 (0.05)			−0.02 (0.03)		P = 0,301*

The data was presented in mean (SD) or median (IQR);

* p < 0.05 (paired t test);

** p < 0.05 (Mann-Whitney test).

After six months, weight (p < 0.001), and height (p < 0.001) had increased, and BMI SDS (p = 0.016) had reduced in the probiotic group. In the placebo group, there was also an increase in weight (p < 0.001), height (p < 0.001), abdominal circumference (p = 0.024) and a reduction in the BMI SDS (p = 0.060), but the differences in these anthropometric measurements were not statistically significant between groups.

In respect of body composition (Table 3), it was found that at the beginning of the study both groups had a high percentage of body fat. In the probiotic group there was a decrease in the percentage of total body fat (p = 0.017) and an increase in lean mass (p < 0.001). In the placebo group, there was a decrease in the percentage of total body fat (p = 0.004), a decrease in trunk fat (p = 0.002) and an increase in lean mass (p = 0.002). However, the variation of these parameters did not differ between groups.

**Table 3:** Body composition of patients using probiotics or placebo, expressed as mean (SD)

	PROBIOTIC (n = 22)			PLACEBO (n = 22)		
	Basal	6 months	p-value	Basal	6 months	p-value
Total Body Fat (%)	47.4 (5.7)	45.8 (6.1)	**P = 0,017** *	47.1 (5.9)	44,9 (6.1)	**P = 0,004** *
Delta Total Body Fat (%)	−1.6 (2.9)			−2.2 (3.1)		P = 0,544*
Trunk Fat (%)	49.2 (6.6)	47.8 (6.5)	P = 0,124*	49.0 (6.4)	46.0 (6.3)	**P = 0,002** *
Delta Trunk Fat (%)	−1.42 (4.2)			−3.0 (4.0)		P = 0,205*
Lean Mass (kg)	35.3 (8.4)	40.1 (8.6)*	**P < 0,001** *	37.0 (8.3)	41 (9.1)	**P < 0,001** *
Delta Lean Mass (kg)	4.8 (3.6)			3.9 (2.5)		P = 0,334*

The data was presented in mean (SD);

* p < 0.05 (paired t test).

When we evaluated the patients according to gender at the beginning of the study, we observed that there was no difference in the BMI SDS, height SDS and lean mass between them, however, the total percentages of body fat, male and female respectively (45.1 ± 6.2 and 48.9 ± 4.9, p = 0.030) and trunk fat (46.4 + 6.7 and 51.2 + 5.5, p = 0.012) were higher in the female. Given the difference in body composition between the males and females, it was decided to separate the analysis by gender.

When we analyze the comparison between the male group we observed a higher reduction in BMI SDS in males of probiotic group (median – 0.26; IQR −0.42 to −0.03) in relation to placebo group (median - 0.03; IQR −0.11 to −0.09, p = 0.05), with no difference in relation to the other parameters analyzed. In respect of the females, there was greater weight gain (probiotic group: median 5.0; IQR 3.4 to 6.8 and placebo: median 2.5; IQ 0.6 to 5.2, p = 0.023), and greater gain in lean mass (probiotic group: median 4.8; IQR 3.7 to 8.4 and placebo: median 3.0; IQR 1.3 to 4.7, p = 0.041) in the probiotic group than in the placebo group. Despite the greater weight gain, there was no difference regarding the variation in the BMI SDS between the groups. There was no difference in BMI SDS change after probiotics or placebo when we compared females and males.

## DISCUSSION

The patients evaluated in this study showed elevated BMI SDS, WC/H ratio and a high percentage of fat mass, suggesting that they were individuals at higher metabolic risk. Some research shows that a high BMI and waist circumference in childhood are associated with an increased risk of metabolic syndrome in adulthood (18,19). Elevated WC and WC/H > 0.5 also reflect the increased visceral fat in this population. We performed an analysis of body composition through DXA and observed a high percentage of total body fat in both groups at the beginning of the study.

Although the participants in the two groups were matched for age and all of them were in puberty, they were not divided according to the Tanner pubertal stages (10). We know that changes in adipose tissue, as well as its distribution during adolescence, are strongly influenced by sexual maturation, and this has been happening earlier, especially in overweight girls (20). However, due to the small number of patients in each group, it was impossible to analyze the data by Tanner criteria. As already described, it was observed that body composition in the females in this study was different to that of the males, with an increase in the percentage of total body and trunk fat. This reinforces the importance of more accurate assessment of body composition and individualization of the therapeutic regimens.

In recent years, there has been an increase in the number of studies affirming the relationship between excess weight and imbalances in the intestinal microbiota, and considering the potential use of probiotics in the treatment of obesity. As previously mentioned, *L. rhamnosus* is one of the most used species in studies for its effects, especially in relation to metabolic syndrome and anti-obesity effects, which have also been reported in many animal studies (21–23). However, studies evaluating probiotic therapy with *L. rhamnosus* in children and adolescents with obesity are scarce. To the best of our knowledge, no study has previously assessed body composition using DXA after therapy with *L. rhamnosus* in these individuals.

After the intervention period, it was noted that there was weight gain in both groups, but with a reduction in BMI SDS. This can be explained by the fact that the patients were still growing. Some studies suggest that a reduction in BMI SDS, even at the expense of increased growth velocity instead of a weight loss, is associated with improved metabolic status in this age group (24).

It is important to highlight that there was no difference in the variation of BMI SDS between probiotic and placebo groups, that is, the probiotic group does not seem to have benefited from supplementation. However, we suggest that this reduction in BMI SDS in both groups may have occurred due to improvements in diet because of the nutritional advice given throughout the therapy. It is important to remember that increased knowledge through nutritional education can change eating practices, leading to changes in BMI (25). Unfortunately, it was not possible to reassess the dietary recall after intervention.

Although the reason is still unknown, a meta-analysis suggested that probiotics may promote weight loss in adults, but weight gain in children, especially when supplemented with probiotics of the *Lactobacillus* species (26). When we analyzed the DXA results, we found that there was no difference in body composition between the groups. However, both showed a reduction in total body fat percentage, trunk fat percentage and an increase in lean mass, which indicates an improvement in the balance of and energy intake and expenditure, with a greater commitment to better nutrition motivated by the monitoring of our team.

A double-blind, randomized, placebo-controlled study of 19 adolescents with obesity, which used a mixture of *Lactobacillus* and *Bifidobacterium* species for 16 weeks, also found an increase in trunk and total adiposity when compared to the placebo group. Jones and cols. (27) also found that multiple probiotic supplementation (S*treptococcus thermophilus*, *Lactobacillus paracasei*, *Lactobacillus plantarum*, *Lactobacillus delbrueckii*, *Bifidobacterium breve* and B*ifidobacterium longum*) increased the adiposity of Hispanic adolescent with obesity; the study found that there was a major increase in total and trunk adiposity. A study by Videhult and cols. (28), evaluated the impact of therapy with another type of probiotic (*Lactobacillus paracasei* ssp.) on the body composition of school-age children without obesity and found that there was no change in growth and modulation of body composition in these individuals.

When patients were divided between genders, important differences were noticed. Comparing placebo and probiotic groups, there was a higher reduction in BMI SDS only in boys of probiotic group. During puberty, it is easier to reduce BMI SDS in male patients, mostly because of testosterone production during this phase. This result could suggest that probiotics could enhance this reduction. Although the percentage of total body fat was high in both groups, after six months the percentage of fat in the trunk remained much higher in the females. We know that during puberty, variations in the percentage of total body fat appear, with differences in the composition and distribution of body fat between genders due to the action of hormones that induce a marked sexual dimorphism. Males are expected to have greater muscle mass gain compared to fat mass gain, and females to have greater fat mass gain (especially in the abdominal region), naturally due to sexual and reproductive development (29). The literature shows that excess body fat can increase the risk of metabolic changes and produce a higher prevalence of risk factors for cardiovascular diseases, including higher concentrations of triglycerides, insulin, leptin, systolic and higher diastolic blood pressure, in addition to reduced high density lipoprotein (HDL-C) (30) which explains the growing concern about obesity in this age group, and the increasing research into effective preventive and treatment solutions. These findings suggest that probably the treatment approach must be different according to gender.

Regarding the information obtained from the patients’ dietary record, although we expected the data to reflect the individual's usual diet in a qualitative and quantitative way, we suspect that patients, in many cases, may have omitted information regarding consumption and food quantities, especially carbohydrates and total fat. Although 24-hour dietary recall is frequently used, the quality of the information depends on the patient's memory and cooperation, which in this age group is a challenge. However, this tool helped us to investigate the individuals’ eating style and to conclude that there were no statistical differences between the two groups’ eating styles, with the participants’ diets appearing to be low in fiber and calcium, and high in cholesterol and sodium. Among the various factors that may explain the inadequacy of the adolescent’ diets, the lack of variety, the excessive consumption of highly processed foods and drinks and the low consumption of fruits and vegetables stand out. Similar results have been found in the analysis of food consumption in this age group in Brazil (31).

We should point out that the present study had some limitations in respect of the sample size, the difficulty in getting the patients to attend the consultations and meet the deadlines for the exams and taking the probiotic/placebo every morning. In addition, we did not measure improvements in diet or physical activity during the study period, which creates a potential bias in the analysis of lean mass gain, for example. It was also not possible to divide the groups according to pubertal staging or degree of obesity because of the small sample size. Unfortunately, it was not possible to get information of nutritional status or education of all parents, so this data was not analyzed.

In conclusion, that supplementation with this strain of probiotic was not effective in promoting weight loss or improving body composition of this population compared to the placebo group, except in pubertal males in the probiotic group, who presented a higher reduction in their BMI SDS. However, the nutritional guidance provided during therapy may have had a favorable impact on BMI, which can be seen positively in this age group. Additional studies are needed with larger sample sizes and long-term follow up to determine the benefits of supplementation with *Lactobacillus rhamnosus*.
